# News and Miscellany

**Published:** 1902-12

**Authors:** 


### News and Miscellany.

**Chambers Visiting List for 1903** is now ready. We have received a copy. It is "perpetual and practical." Price $1.00 postpaid. Get the best.

**Personal.**—Dr. G. Frank Lydston, of Chicago, has just returned from an extended tour of Australia, New Zealand, Samoa and the Hawaiian Islands.

**The New Orleans Polyclinic** opened for its sixteenth annual session on November 3, 1902. Up to date the class is large and the prospects are brilliant for a very successful term.

---

**Sharpe & Dohme,** to facilitate the delivery of their products and better serve their southern and western trade have established a depot at 119 S. Fourth street, St. Louis, and will carry a complete stock and make prompt shipments.

---

**Dr. E. E. Guinn,** Secretary of the East Texas Medico-Chirurgical Society and late physician to the Rusk penitentiary (resigned Nov. 17, 1902) has returned to his old home in Jacksonville, where he will resume the general practice of medicine, giving special attention to electro-therapeutics.

---

**Dr. A. B. Kennedy** of Bonham, Surgeon 2nd Brigade, T. V. Guard, has been promoted to Medical Director of the Division, with the rank of Lieutenant Colonel, vice Col. F. C. Ford (Nacogdoches), retired, and Capt. B. V. Ellis of Paris, Surgeon 3rd Infantry, is promoted to Surgeon of 2nd Brigade, vice Kennedy.

---

**New Orleans Polyclinic.**—Sixteenth annual session opens November 3, 1902, and closes May 30, 1903. Physicians will find the Polyclinic an excellent means for posting themselves upon modern progress in all branches of medicine and surgery. The specialties are fully taught, including laboratory work. For further information address New Orleans Polyclinic, Postoffice Box 797, New Orleans, La.

---

**Dr. Charlton,** Rockefeller Fellow in Pathology of McGill University, obtained some remarkably good results in the treatment of scarlet fever with Stearns' antistreptococcic serum, securing thirteen recoveries in fifteen cases and what is considered by him still more remarkable, an almost immediate change from a serious condition to a very mild one after the serum was given. A reprint of Prof. Charlton's paper from *Montreal Medical Journal* may be had free from Frederick Stearns & Co., Detroit, Mich.

---

**State Health Officer** George R. Tabor is in Washington, D. C., to represent the State of Texas at the convention of the Pan-American Sanitary Commission. The purpose of this meeting is to devise ways and means to prevent contagious diseases from being transmitted from one country to another.

This gathering of representatives of the various countries will be a most important one, especially those from South and Central America, as many of the worst contagious diseases originate in these countries.

---

**Death of Dr. Gardner.** Dr. Asa B. Gardner, of Bellville, late President of the Texas State Medical Association, died at Mineral Wells, Texas, of Bright's disease, Oct. 26th, 1902. Dr. Gardner graduated from Bellevue Hospital Medical College, New York City, in 1881. He held several important positions in his section as railroad surgeon, insurance examiner, etc. Dr. Gardner's untimely death—he was scarcely past 40—is much lamented. He was a fine physician and a genial and very popular man, greatly esteemed by his colleagues.

---

**Death of Dr. Thorpe.**—We are pained to announce the death of Dr. H. W. Thorpe, of Liberty Hill. He was a most estimable gentleman and an ethical and true physician, a real "old Virginia gentleman of the old regime." He was born in Winchester, Va., 1844, spent his younger days at Elkton, Maryland, where he acquired his literary education, graduated at the Baltimore City Medical College in 1870, lived in Minnesota one year, then came to Houston, Texas, where he lived 5 years before going to Liberty Hill, Texas, where he practiced his profession up to the day of his death, having passed away November 6th, last, age 58 years.

---

**A Solid Gold Spike!** A solid 14-karat gold spike will be driven at a point between Italy and Ft. Worth early in December, with appropriate ceremonies, which will mark the completion of the Fort Worth division of "The Texas Railroad" the I. & G. N., between Fort Worth and Galveston. This new line is now in operation between Italy, Mertens, Waco, Marlin, Calvert, Bryan, Navasota, Houston and Galveston, and will be ballasted and in operation between Italy and Fort Worth early in January, thus forming a great trunk line between North and South Texas, as well as between North, and Southwest Texas and Mexico. Construction on the new line batween Navasota and Madisonville, via Anderson is being pushed rapidly.

---

**A New Dental School**—The Dallas Medical College announces the organization of a department of dentistry to be opened about Jan. 1st. The equipment is now being purchased and will shortly be installed. We have been watching the development of the Dallas Medical College for some time and have been both pleased and surprised at the splendid record made. The school is now in its third year, has a salaried faculty, splendid laboratories and clinics and 150 students in attendance. The fees are somewhat higher than is customary in the Southwest, but with the advantages offered, the charges are very reasonable. We take pleasure in congratulating both the trustees and faculty on the excellent work done.

---

**Diabetes Mellitus:** Its Treatment and Cure. Clinical reports by Drs. Stuckey, Dixon, Sterne, Hickey, Haines, Bishop, Goldstein, *et al.*

We have received a neat pamphlet bearing the above title, sent us by the Charles Roome Parmele Co., manufacturers of "Arsenauro," "Mercauro," etc. The reports are a high testimonial to the value of Arsenauro, and our readers will do well to write to Parmele Co., 45 John street, New York, for a copy of the pamphlet. The text books do not give much encouragement to the physician when speaking of diabetes mellitus, and the experience of such distinguished practitioners as the contributors to the pamphlet will have peculiar interest and value to our readers. The Chas. Roome Parmele Co. work along most strictly ethical lines. They advertise to and deal only with the physician, refusing even to send literature to any other. Send for a copy of the pamphlet, Doctor, and read it.

---

**A very high compliment to "The Texas Railroad."**—"In a business letter to General Passenger Agent D. J. Price, of the International & Great Northern system of Texas, on my return from Europe, I was pleased to remark that I had not run across any railway in Britain, Germany, Russia, Switzerland, Italy or France that excelled the I. & G. N. in the solidity and smoothness of its track, or the beauty and comfort of its new coaches—the company's own make at its Palestine shops. "If you had said it in *The 400*," replied Mr. Price, "we should have considered it a very notable compliment." I am very glad to repeat it in *The 400*, and also to reiterate what I said of the I. & G. N. equipment in the May number—that it is not surpassed and seldom equalled by the older and richer railroads in the North and East. This is remarkable, in view of the age and population of Texas, but I claim to be a judge."—"*Chicago 400*," *November, 1902.*

---

**American Public Health Association.**—About the time the "Red Back" is being read by its friends, the American Public Health Association will be in session in New Orleans (December 8 to 12, inst.) The Executive Committee has selected the following topics for consideration: The Pollution of Public Water Supplies. The Disposal of Refuse Material. Animal Diseases and Animal Food. Car, Steamship and Steamboat Sanitation. Etiology of Yellow Fever. Demography and Statistics in Their Sanitary Relation. Cause, Prevention, Period of Incubation and Duration of Infectious Diseases. Public Health Legislation. Cause and Prevention of Infant Mortality. Disinfectants and Disinfection. National Leper Homes. Dangers to the Public Health from Illuminating and Fuel Gas. Transportation of Diseased Tissue by Mail. The Teaching of Hygiene and Granting of Diploma of Doctor of Public Health. Sanitary Aid Societies. The Relative Immunizing Value of Human and Bovine Vaccine Virus. The Investigation of the Canteen System of the United States Army.

---

**Washington Post-Graduate Medical School**—Believing that the National Capital presents many special advantages for post-gradutae instruction, the leading members of the profession in Washington City have organized a Post-Graduate Medical School. The course of instruction will consist principally of clinics at the different hospitals of the City and of practical lab oratory work. One or two didactic lectures will also be given daily upon such branches as Preventive Medicine, Military Medicine and Surgery, Preventive Inoculations, Serum Therapy, etc.

General Geo. M. Sternberg, U. S. A., has been elected president of the faculty and the Surgeon Generals of the Army, Navy, Public Health and Marine Hospital Service are members of the Executive Committee.

It is believed that the courses of instruction will be especially valuable for physicians who contemplate entering one of the branches of the public service, also for a general practitioner and for those who intend to practice surgery, gynecology, ophthalmology or laryngology as a specialty. The special attention given to Preventive Medicine and to laboratory work in Bacteriology and Sanitary Chemistry will afford public health officers unusual advantages for perfecting themselves in the scientific studies which serve as a foundation for their particular work.

The first session of the school will commence on Monday, January 12th, 1903. Circulars containing full information will be sent upon application to the President of the Faculty, General Geo. M. Sternberg, U. S. Army, 2144 California Avenue, Washington, D.C.

---

**Resolutions** in reference to the conduct of the health authorities of California in dealing with the plague situation, adopted by the Conference of State and Provincial Boards of Health of North America at the annual meeting held at New Haven, October 28-29, 1902. "Whereas, bubonic plague has been present in California since March, 1900, information as to the extent of the disease being withheld by the local authorities, no effective measures of restriction having been put into operation, and the history of the outbreak, so far as we can ascertain from authoritive sources being as follows: [Detailed report of cases omitted; 88 in all.—Ed.] And, whereas, thirty of these cases have occurred since July 13th, 1902, no information as to their origin or exact location having been furnished, no effective steps having been taken to restrict the spread of the disease, the City Board of Health of San Francisco being helpless, and the *mala fides* of the State Board of Health of California having been fully established by the foregoing history, supported by documentary evidence in the possession of this Conference; therefore, be it Resolved, that the Conference of State and Provincial Boards of Health of North America views with abhorrence the irretrievable disgrace of the present State Board of Health of California, and pronounces the plague situation in California a matter of grave national concern; and, be it further Resolved, that the National Conference of State and Provincial Boards of Health of the United States consider the propriety of calling upon the Surgeon-General of the United States Public Health and Marine Hospital Service to arrange at the earliest possible date a joint conference for the purpose of eradicating plague from the United States."

A very just rebuke. The disgraceful and dangerous situation emphasizes the pressing need of a Commissioner of Public Health in the Cabinet.—Ed.

